# EFFECTIVENESS OF CEMENTLESS PROSTHESIS IN PATIENTS OVER 70 YEARS OF AGE

**DOI:** 10.1590/1413-785220253302e287096

**Published:** 2025-06-02

**Authors:** Vinicius Borges Pires, Vinicius Ferreira Pires Bueno, Nivaldo Fernandes, Tarciso Liberte Romão Borges, Leandro Alves de Oliveira

**Affiliations:** 1 Universidade de Rio verde (UniRv), Goias, GO, Brazil.; 2 Hospital Estadual de Urgências Governador Otávio Lage de Siqueira (HUGOL), Goiania, GO, Brazil.; 3 Faculdade Morgana Potric, Goias, GO, Brazil.; 4 Hospital Estadual de Aparecida de Goiânia Cairo Louzada (HEAPA), Goiania, GO, Brazil.; 5 Universidade Federal do Tocantins (UFT), Tocantins, TO, Brazil.; 6 Universidade Federal de Goiás (UFG), Hospital das Clinicas, Goiania, GO, Brazil.; 7 Clínica de Ortopedia e Traumatologia (COT), Goiania, GO, Brazil.

**Keywords:** Hip Replacement Arthroplasty, Aged, Treatment Outcome, Artroplastia de Substituição de Quadril, Idoso, Resultado do Tratamento

## Abstract

**Introduction:**

Hip replacement in the elderly is challenging due to their unique clinical conditions. Choosing the prosthesis requires an evaluation of risks and benefits, considering the implant's durability, postoperative recovery, and patient longevity. Uncemented prostheses have emerged as a viable alternative, offering osseointegration, as well as mechanical and biological stability.

**Methods:**

A retrospective observational study analyzed the medical records of patients aged 70 years or older who underwent total hip arthroplasty (THA) with an uncemented prosthesis between 2013 and 2022. Age, sex, diagnosis, procedures performed, and postoperative follow-up time were evaluated.

**Results:**

Of the 231 patients analyzed, women predominated (62%), with an average age of 78.5 years for men and 79.1 for women. There was a consistent preference for the uncemented technique across all age groups, with less than 20% of cases requiring cerclage. Complications were minimal, with over 90% of cases being complication-free, highlighting the technique's efficacy.

**Conclusion:**

The technique reduces complications, including inflammatory reactions and long-term bone loss. The low rate of surgical revision and return to recreational activities reinforce its efficacy. Although cemented prostheses have historically been preferred, uncemented prostheses offer advantages in the elderly population, as they preserve bone and facilitate revisions. ***Level of evidence III; Therapeutic Studies - Investigation of Treatment Outcomes.***

## RESUMO

**Introdução:**

A substituição do quadril em idosos é desafiadora devido às suas condições clínicas singulares. A escolha da prótese requer avaliação de riscos e benefícios, considerando a durabilidade do implante, a recuperação pós-operatória e a longevidade do paciente. As próteses não cimentadas surgiram como alternativa viável, oferecendo osseointegração e estabilidade mecânica e biológica.

**Métodos:**

Estudo retrospectivo observacional, analisando prontuários de pacientes com mais de 70 anos submetidos à artroplastia total de quadril (ATQ) com prótese não cimentada entre 2013 e 2022. Foram avaliados idade, sexo, diagnóstico, procedimentos realizados e tempo de acompanhamento pós-cirúrgico.

**Resultados:**

Dos 231 pacientes analisados, predominaram mulheres (62%), com média de idade de 78,5 anos para homens e 79,1 para mulheres. Houve uma preferência consistente pela técnica não cimentada em todas as faixas etárias, com menos de 20% dos casos requerendo cerclagem. Complicações foram mínimas, com mais de 90% dos casos sem complicações, destacando a eficácia da técnica.

**Conclusão:**

A técnica reduz complicações como reações inflamatórias e perda óssea a longo prazo. A baixa taxa de revisão cirúrgica e retorno à atividade recreativa reforçam sua eficácia. Embora as próteses cimentadas tenham sido historicamente preferidas, as não cimentadas oferecem vantagens na população idosa, preservando osso e facilitando revisões. ***Nível de Evidência III; Estudos Terapêuticos - Investigação dos Resultados do Tratamento.***

## INTRODUCTION

The choice of prosthesis type used in hip replacement surgeries for the elderly requires careful evaluation of risks and benefits, considering the implant's durability, postoperative recovery, and patient longevity.^1^ Surgery relieves pain and improves quality of life, especially in the elderly with osteoarthritis. With the increase in longevity, the number of hip replacements is rising. Uncemented prostheses are a viable alternative to cemented ones, benefiting patients over 70 years old.^2^

Uncemented prostheses are popular due to their osseointegration, which provides greater mechanical and biological stability, better load transfer, and reduced aseptic loosening, crucial factors for the longevity of the implant in elderly patients with extended life expectancy.^3^

Bone quality compromised by osteoporosis and anatomical deformities can complicate the fixation and stability of the implant. The choice of size and design for the prosthesis should consider individual anatomical characteristics to ensure a proper fit and minimize complications.^4^

By analyzing clinical outcomes, revision rates, and complications in patients over 70 years old, the aim is to inform treatment decisions and promote better outcomes in hip replacement for the elderly.

## MATERIALS AND METHODS

The study was approved under CAAE No: 80917524.7.0000.0237. All participants signed the informed consent form (ICF).

This retrospective observational study was conducted using data collected from electronic medical records spanning the years 2013-2022 in a private clinic located in a Brazilian capital city. The following were analyzed: age, sex, laterality, injury diagnosis, procedures performed, and time elapsed between diagnosis and revision surgery.

Patients who were at least 70 years old at the time of their surgery, who underwent hip arthroplasty using an uncemented hip prosthesis, who had the surgery in a private clinic located in a Brazilian capital city, and who have medical follow-up in the same hospital for at least 6 months post-surgery for post-operative supervision were selected. Patients who did not meet the sociodemographic criteria of the study, who abandoned treatment, or who failed to comply with the established follow-up criteria for the study (i.e., loss to follow-up) were excluded.

## RESULTS

**Figure 1:**
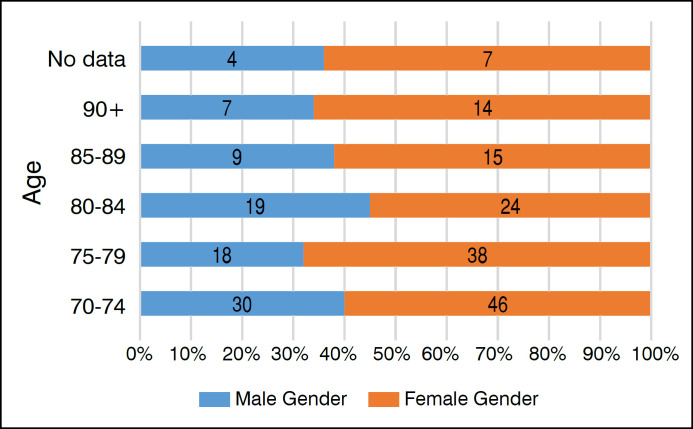
Gender by age group.

A total of 144 female patients and 87 male patients were identified, aged between 70 and 107 years, with an average age of 78.5 years for men and 79.1 years for women, a global median of 77 years, and a standard deviation (σ) of 7.1 years. According to the data presented in Table 1, there was a general predominance (62%) of the female sex, and as shown in Figure 1, the predominance remains consistent across all age groups evaluated. For all statistics involving the study parameters, patients without data were excluded from the analysis.

**Table 1:** Gender predominance.

Gender	Sample	Percentage
Female	144	62.33%
Male	87	37.66%
Total	231	100%

The data obtained are consistent with the epidemiology of patients affected by the main indications for THA, including femur fracture and hip osteoarthritis.

**Figure 2:**
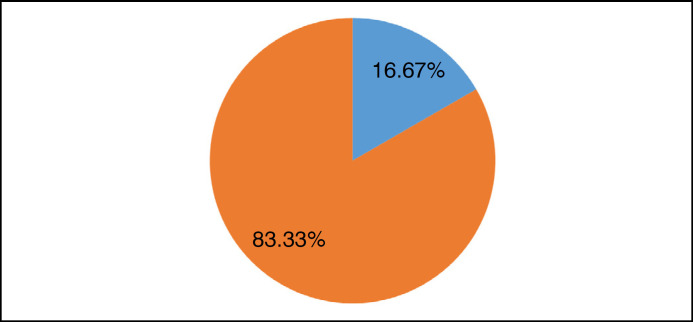
Distribution by method of patients undergoing cerclage.

The sampling was divided between patients undergoing cemented THA and non-cemented, regardless of laterality or gender. Patients who were bilaterally prosthetized or who received hybrid fixation techniques (cemented and non-cemented prostheses) were also included among the surgeries. Figure 2 shows a heterogeneous distribution of the techniques employed; however, there is a consistent predominance of the non-cemented technique across all age groups.

Of the patients in this group who met the inclusion criteria, only 13.51% underwent dissimilar techniques. With the available data, it was not possible to establish a direct correlation between the indication of alternating techniques in the same patient and other parameters, given the homogeneous distribution of these patients in gender, age group, and the proximity of the THA dates.

Among the patients in whom the technique was repeated, non-cemented prosthesis stood out at 75.7%. Hybrid techniques were not described in the sampling. In 13 patients, it was necessary to perform cerclage concurrently with THA, and, as indicated in Figure 2, less than 20% of the cases involved cemented prostheses.

Due to the known correlation between chronological age and bone fragility, the reduction of fixation is a significant obstacle to consider when selecting a prosthesis.

There is a clear trend towards a progressive increase in the use of non-cemented prostheses over time. Contrary to the observations of John Chanrley, a pioneer in the development of the first joint prostheses in the 1960s, who concluded in his studies conducted over 50 years ago that the success of the surgery was closely linked to an effective cementation technique.

**Figure 3:**
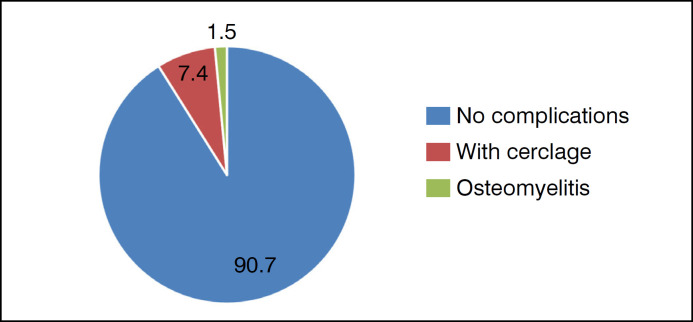
Complications in patients undergoing uncemented THA.

Figure 3 illustrates the complications observed in patients undergoing uncemented THA.

It is noted that in more than 90% of the analyzed cases, no complications were observed, thus highlighting the effectiveness of the cementless technique.

Despite various modifications to THA techniques over the decades, cementation remains a benchmark standard. It is essential to emphasize that the advancements aimed primarily to minimize complications arising from the interaction of the cemented material with the bone; this interaction does not exist in the uncemented technique, avoiding possible allergic, inflammatory, or infectious reactions. Preventing bone degradation and reducing the chances of needing surgical revisions.

## DISCUSSION

Over the last 20 years, cementless prostheses have gained significant ground in most Brazilian orthopedic centers, as observed in the clinic where the study was conducted. However, the discussion regarding the possible superiority of one of the techniques still lacks a definitive conclusion.

The choice of a qualitative-quantitative approach to analyze the research data was based on the need to understand both quantitative and qualitative aspects related to total hip arthroplasty (THA). This allowed for a more detailed analysis of the variables involved, including not only numerical and statistical data but also qualitative considerations regarding surgical methods, patient characteristics, and trends over time.

The selection of a sample of 231 patients aged 70 years and older who underwent THA between 2013 and 2022 was motivated by the need to obtain a representative and specific sample for the study. This demographic group was chosen due to the clinical relevance of surgical interventions on the hip in elderly patients, considering the prevalence of conditions such as femoral fractures and osteoarthritis in this age group.

The observed age range and sex are consistent with the data obtained by Brüggemann et al.,^1^ who analyzed cases treated from 1987 to 2020 in Norway. In this study, the cemented technique was used in 61% of the surgeries, but the uncemented technique gained popularity starting in 2008. In addition to this similarity, the study by Brüggemann et al.,^1^ observed that only 1% of the cases presented intraoperative periprosthetic femoral fracture, a remarkably low percentage considering the study period and the number of patients included. These results clearly relate to those obtained in this study.

The increased risk for uncemented stem fixation versus cemented stem fixation was relatively stable across different age and sex strata; thus, the uncemented total hip arthroplasty (THA) technique presents better prognoses, especially when considering the interaction of the material with the patient's body.

There is a heterogeneous distribution of the techniques employed, with a consistent predominance of the uncemented technique across all age groups. Even among patients aged 90 years or older, the preference for uncemented prostheses reaches 69%. The study by Delmonte et al.,^3^ analyzes the effectiveness of uncemented hip prosthesis in managing hip dysplasia; in this study, about 86% underwent uncemented THA surgery between 1999 and 2021, with patients aged between 26 and 77 years. Consolidation of all femoral osteotomies was noted, with no delays in consolidation or pseudarthroses observed in any patient. Delmonte et al.,^3^ present concurrent results with those obtained in this study, with a low incidence of the need for re-surgical intervention (one patient).

Another study that also reports on uncemented THA intervention in managing rheumatoid arthritis was conducted with a sample of 24 patients; throughout the study development period, no cases of prosthesis loosening were evidenced, nor was there vertical migration of the stem, only one case of distal perioperative fracture at the calcaneus, treated with cerclage, without affecting the quality of clinical and radiographic results for the patient, and no cases of dislocations or infections were reported. In the clinic, the need for additional procedures, such as cerclage, was relatively low, with only 16.67% of cases requiring cerclage.^4^

Patients over 80 years in the sample were significant (88 patients, among both women and men); the study by Yuasa et al.^5^ analyzed the results of uncemented total hip arthroplasties (THA) in patients aged 80 years or older, where none of the THAs required a re-surgical approach due to poor initial fixation or early loosening. In this study, conducted in Japan, all analyzed patients were female; this age group is associated with a known history of greater bone fragility due to age and reduced calcium absorption after menopause.

The overall predominance (62%) of females in the sample corroborates the epidemiology of indications for THA, such as femur fracture and hip osteoarthritis. This trend remains isolated across all age groups evaluated. Talking with other studies.^6,7^

Solarino et al.,^6^ highlight that men and women have significant anatomical differences in the hip, which may influence the results of THA. Women tend to have a wider pelvis and a larger femoral neck angle, which can affect the biomechanics of the prosthesis and the wear of the components. The authors do not present the percentage data for each sex; however, it is noted that women tend to be more prevalent due to the higher incidence of osteoarthritis and osteoporosis, conditions that often lead to the need for THA. The study by Liu et al.,^7^ compares the long-term results of patients undergoing total hip arthroplasty (THA) using cemented versus uncemented femoral components. The study reveals a higher revision rate in the group with uncemented components (5.2%) compared to the group with cemented components (3.8%). This difference was not statistically significant. Patients with cemented components had a higher incidence of perioperative complications, such as pulmonary embolism and intraoperative fractures, compared to the uncemented group. It is emphasized that uncemented prostheses are designed to allow direct bone growth on the surface of the implant, promoting a more natural and solid integration. This osseointegration can lead to greater long-term stability and reduce the need for revisions. Liu et al.^7^ emphasize that cementing can lead to bone necrosis around the cement, compromising bone quality in the long term. Uncemented prostheses better preserve bone structure, facilitating possible revision procedures in the future.

Following the technique chosen to develop the case study at the Clinic, the study by Li et al.^8^ (2020) shows better results in procedures where the uncemented technique was employed. A total of 8 studies involving 1,577 hips (782 uncemented and 795 cemented) were included in this meta-analysis. The meta-analysis indicates that the operation time for cemented hemiarthroplasty was longer than for uncemented hemiarthroplasty. This study notes that the cemented technique resulted in a longer hospitalization time and a higher rate of pulmonary embolism. It is also emphasized that the uncemented procedure has a shorter operating room time.

A study conducted in Europe has shown that, in recent years, there has been an increase in the use of uncemented prostheses among the elderly population. This study highlighted trends in the use of uncemented fixation, particularly in patients over 75 years old. The study included countries such as Australia, Denmark, England and Wales, Finland, the Netherlands, New Zealand, Norway, Romania, Sweden, and Switzerland, all of which have high completeness rates. Through this study, it was noted that in Scandinavian countries, the percentage of total hip arthroplasties (THAs) performed with uncemented fixation gradually increased, with a notable increase in the use of uncemented fixation in patients over 75 years old.^9^ It was again observed that there was a shorter hospitalization time and fewer postoperative complications.

That said, the uncemented technique still showed a clear advantage in the study when used in men up to 75 years old. Stabilization and lower fall rates in patients aged 75 or older were also attributed; however, the hypothesis of better awareness methods is raised.^9^ Rassir et al.,^10^ (2021), when analyzing THAs performed with bone cement, discuss a study on the bone cement implantation syndrome (BCIS), which is characterized by hypoxia, hypotension, and loss of consciousness during cemented THA and can result in death. This study also reports that BCIS occurred in 26% of arthroplasties in general, and the incidence in hemiarthroplasties was 31%. Specifically, in hip hemiarthroplasties, a 28% occurrence of BCIS was recorded, which is a relatively high percentage considering the study population. In surgical re-approach procedures, BCIS was present in 20% of cases. Among patients who experienced severe BCIS, a higher probability of death was noted within 30 days after the procedure. Among the factors independently associated with the development of severe BCIS, age over 75 years and chronic renal failure were the most prevalent specifics. The uncemented technique does not use surgical cement. Thus, this serious complication is completely ruled out as a possible occurrence.

The study by Jämsen et al.^11^ shows that periprosthetic fracture was the primary mode of failure for uncemented hip replacements, indicating that the most serious complication observed could be treated with cerclage, a relatively straightforward procedure. After 1 year, there were no differences in survival rates, once again showing favorable patterns for the cementless technique.

Zimmerer et al.^12^ conducted a cohort study with 96 patients aged between 76 and 84 years who underwent cementless total hip arthroplasty (THA), with 79 patients completing a comprehensive questionnaire on postoperative recreational activities, including sports. After surgery, 71% of patients were active in at least one recreational activity, 72% of patients returned to recreational activities within one month after surgery, and 26% resumed sports activities within three months. The main findings of the study are that the vast majority of patients could return to any type of recreational behavior after cementless THA.

Finally, the study by Yuasa et al.,^6^ reports that among the 30 cementless THAs performed in patients over 80 years old, no patient required surgical revision due to poor fixation or early loosening of the prosthesis. Thus, it was possible to conclude in this context that cementless THA is safe and durable in this population.

## CONCLUSION

Cementless prostheses offer greater bone preservation and facilitate revision procedures, when necessary, which can be beneficial for individuals facing mobility issues and fragile health. The hip arthroplasty procedure is significantly safer than other techniques, as demonstrated in our analysis of the elderly population and that of other authors. The surgical revision rate is expressively low, comparable to the same procedure frequently performed in the United States of America, where the revision rate is only 1%. Thus, considering the benefits in terms of longevity and quality of life, cementless prostheses emerge as a highly advantageous option for this age group.
